# Railway Tunnel Clearance Inspection Method Based on 3D Point Cloud from Mobile Laser Scanning

**DOI:** 10.3390/s17092055

**Published:** 2017-09-07

**Authors:** Yuhui Zhou, Shaohua Wang, Xi Mei, Wangling Yin, Chunfeng Lin, Qingwu Hu, Qingzhou Mao

**Affiliations:** 1 China Railway Eryuan Engineering Group Co., Ltd., Chengdu 610031, China; zhouyh@ey.crec.cn (Y.Z.); meixi@ey.crec.cn (X.M.); lincf@ey.crec.cn (C.L.); 2 School of International Software, Wuhan University, No. 129, Luoyu Road, Wuhan 430072, China; 3 School of Remote Sensing and Information Engineering, Wuhan University, No. 129, Luoyu Road, Wuhan 430072, China; yinwanling1989@126.com; 4 State Key Laboratory of Information Engineering in Surveying, Mapping and Remote Sensing (LIESMARS), Wuhan University, No. 129, Luoyu Road, Wuhan 430072, China; qzhmao@whu.edu.cn; 5 Key Laboratory for National Geographic Census and Monitoring, National Administration of Surveying, Mapping and Geoinformation, Wuhan University, No. 129, Luoyu Road, Wuhan 430072, China

**Keywords:** mobile laser scanning (MLS), point cloud, railway tunnel clearance, clearance coordinate system, tunnel cross section

## Abstract

Railway tunnel clearance is directly related to the safe operation of trains and upgrading of freight capacity. As more and more railway are put into operation and the operation is continuously becoming faster, the railway tunnel clearance inspection should be more precise and efficient. In view of the problems existing in traditional tunnel clearance inspection methods, such as low density, slow speed and a lot of manual operations, this paper proposes a tunnel clearance inspection approach based on 3D point clouds obtained by a mobile laser scanning system (MLS). First, a dynamic coordinate system for railway tunnel clearance inspection has been proposed. A rail line extraction algorithm based on 3D linear fitting is implemented from the segmented point cloud to establish a dynamic clearance coordinate system. Second, a method to seamlessly connect all rail segments based on the railway clearance restrictions, and a seamless rail alignment is formed sequentially from the middle tunnel section to both ends. Finally, based on the rail alignment and the track clearance coordinate system, different types of clearance frames are introduced for intrusion operation with the tunnel section to realize the tunnel clearance inspection. By taking the Shuanghekou Tunnel of the Chengdu–Kunming Railway as an example, when the clearance inspection is carried out by the method mentioned herein, its precision can reach 0.03 m, and difference types of clearances can be effectively calculated. This method has a wide application prospects.

## 1. Introduction

The tunnel clearance is the space required for the safe operation of a locomotive along a fixed track, a spatial clearance free of any barrier within a certain width and height scope, to ensure the normal operation and safety of various types of traffic in the tunnel [1,2,3,4,5]. The most commonly used method for detecting railway clearance—the section detection method, can be divided into two categories, namely, contact type and non-contact type. In early days, contact measurement method was widely applied at home and abroad [6,7,8,9]. Through a framework where multiple extendable arms can be mounted on the flat car, when the flat car is running, all arms contact the tunnel walls, and the sensors on the arms will convert the mobile signals to electrical signals to draw out the transverse and longitudinal sections for different positions. This equipment is relatively cheap compared with manual measurement and the measurement precision can be less than 30 mm, but the measuring speed is slow, only 10 km/h. Using this method to perform the section measurement will interfere with the train traffic on the busy main line, and cannot be applied to electrified sections [6,10,11].

In 1980, the Austrian company Plasser developed the GCM-10 clearance inspection car, with a maximum measuring speed of 18 km/h, where the laser radar ranging principle was applied. In 1983, a 3 km/h clearance inspection car has been developed in France, which used an argon laser light as the source, used a laser beam for scanning tunnel sections, and took photos for the laser belt at the preset laser height [11,12,13,14,15,16,17].

After the 1980s, to solve the detection problems on electrified sections, the non-contact detection method came into being. The Swiss company Amberg Technologies carried out studies for the project, connecting a laser tunnel section measurement instrument to a computer. The computer was used to control and achieve the functions of the section measurement instrument and display the measured section shape. This paved a new way for the future tunnel section measurement methods [11,12,13,14]. Many countries have developed tunnel clearance inspection cars. In 1986, a TV camera clearance inspection car was launched in the UK, which applied the double triangle relationship measurement principle to obtain any 5 m comprehensive profile with a speed up to 100 km/h [18,19,20]. In late 1990s, China developed the SJC-1 tunnel inspection car, which acquired a comprehensive section size at an interval of 5 m at 70 km/h [21,22,23,24]. In early 21st century, the BJSD-2 laser clearance inspection instrument has been developed, which can acquire the section in perpendicular to the tunnel middle line [25,26]. Hu et al., proposed a railway clearance inspection method based on visual perception. Japan has developed a clearance inspection car mounted with CCD sensors [4]. With the development of CCD sensor technology, video compression technology and image processing technology, computer video measurement technology has been increasingly applied to tunnel clearance inspection tasks, and the clearance inspection has been developing in a faster and more precise manner [27,28,29,30,31,32,33].

With the development of 3D laser technology, image processing technology and intelligent robot technology, the multi-sensor integrated 3D laser mobile scanning system runs at a speed of 120 km/h to obtain high density and high precision laser point clouds of tunnels for clearance inspection, which has become the current trend in railway clearance inspection. This method has to tackle with some issues such as what efforts should be made to quickly and automatically carry out the clearance intelligent analysis and calculation based on the 3D laser point cloud [3,34,35,36,37]. For the large volume of point cloud data from laser scanning systems, how to deal with the point cloud to calculate different clearance is still a big challenge.

In view of the problems existing in the traditional tunnel clearance inspection methods, such as low density, slow speed and a lot of manual operations, this paper proposes a tunnel clearance inspection method based on a mobile laser scanning 3D point cloud. First, a dynamic coordinate system for railway tunnel clearance inspection has been proposed. A rail line extraction algorithm based on 3D linear fitting is implemented from the segmented point cloud to establish a dynamic clearance coordinate system. Second, a method to seamlessly connect all rail segments based on the railway clearance restrictions, and a seamless rail alignment is formed sequentially from the middle tunnel section to both ends. Finally, based on the rail alignment and the track clearance coordinate system, different types of clearance frames are introduced for intrusion operation with the tunnel section to realize the tunnel clearance inspection. By taking the Shuanghekou Tunnel of the Chengdu–Kunming Railway as an example, when the clearance inspection is carried out by the method mentioned herein, its precision can reach 0.03 m, and different types of clearances can be effectively calculated. This method has a wide application prospects.

## 2. Methodology

**Figure 1:**
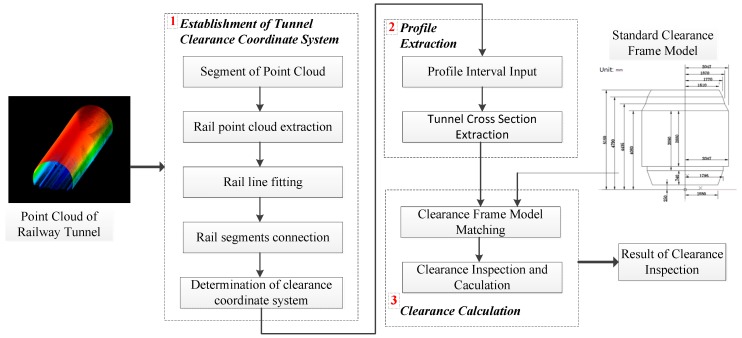
Technical flowchart of the proposed tunnel clearance inspection method.

A mobile 3D laser scanning system (MLS), which integrates a high performance laser scanner, high precision positioning and navigation system typically includes a Global Navigation Satellite System (GNSS) receiver, CCD cameras, control board and industry computer on a vehicle, is used to obtain laser scanning data, positioning and navigation data and image data synchronously. After high positioning and navigation data fusion processing, it can provide accurate position and attitude data for the laser scanner to obtain high density and high precision 3D laser point cloud of the railway track and surroundings [38,39,40]. Based on the processing and analysis of 3D laser point cloud, the rail alignment and tunnel section, different types of clearance frames are introduced for 3D space operations to inspect the railway tunnel clearance. The overall technical process is shown in Figure 1.

In Figure 1, the railway tunnel point cloud data obtained by using the MLS are pre-processed into segments along the line direction, to ensure that all rail segments are linear. Second, the straight rail segments are extracted from each section. The plane where the two centerlines of the left rail and right rail are located, is used to determine the clearance measuring coordinate system. Then, the tunnel cross section baseline is determined by relying on the clearance measuring coordinate system, and cross sections are collected from the tunnel point cloud. Finally, based on the railway tunnel testing rack model, the distance between the tunnel cross section and testing rack is calculated to realize the clearance inspection.

### 2.1. Clearance Coordinate System and Segments of Tunnel Point Cloud

**Figure 2:**
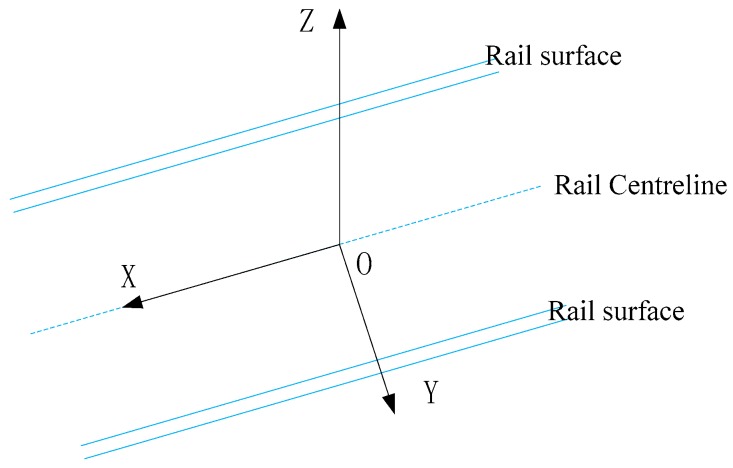
Definition of clearance coordinate system.

The tunnel clearance is a limited cross section profile perpendicular to the centerline of the alignment [3,41,42,43]. The centerline of the alignment should be obtained first in the tunnel clearance inspection, and then a clearance inspection coordinate system should be established. The clearance coordinate system is a local coordinate system, which is determined based on the centerline of the alignment and the rail surface [1,3,41,42,43], as shown in Figure 2. The *X* axis is the centerline of the rail surface, i.e., the central line of the rail. *Y* axis is perpendicular to the *X* axis, and *Z* axis is perpendicular to the rail with the rail surface facing upward.

Therefore, the clearance measuring coordinate system is established on the basis of the determination of the rail straight line. The railway tunnel structure is long and narrow, and the rail applied in the alignment is either linear or curved. However, to ensure that the locomotive will not turn over due to the excessive centripetal force when it is making a turn, the curvature of the curve rail is very small, and the rail in the small length range is almost linear, so this paper focuses on the pre-treatment of the tunnel point cloud data by segments along the line, to ensure that each segment of the rail is linear, and that each can establish a clearance measuring local coordinate system defined by a rail straight line, to carry out the tunnel clearance inspection. In this paper, the tunnel point cloud is segmented at an interval of 5 m.

**Figure 3:**
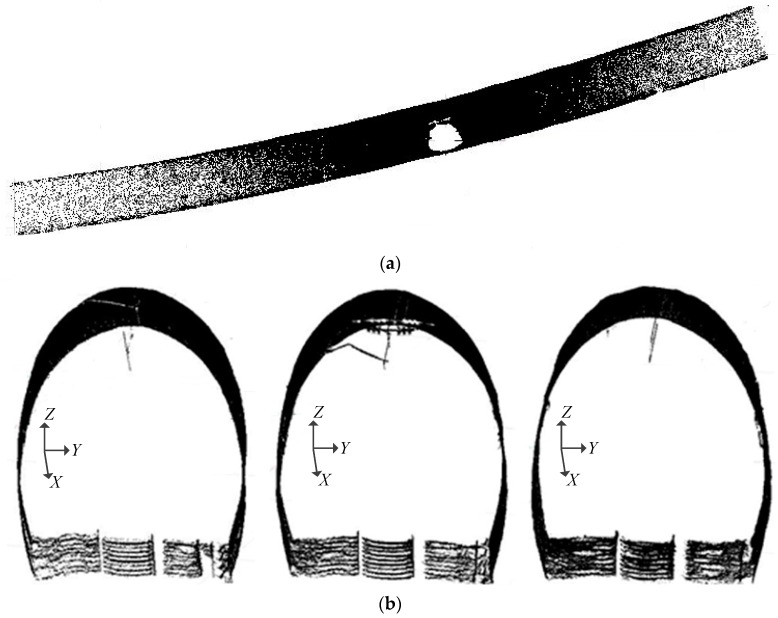
Segmentation results of tunnel point cloud. (**a**) Top view of whole tunnel point cloud; (**b**) point cloud of three segments.

Figure 3 is the effect drawing of segments. From the top view of overall tunnel point cloud shown in Figure 3a, this alignment is curved. After the segmented pretreatment along the alignment, the results of three representative segments are shown in Figure 3b.

After the point cloud segmentation, the rail point cloud extraction and fitting can be made for segments, so that each clearance measuring coordinate system can be defined in accordance with the clearance inspection points. Then, based on this coordinate system, the cross sections in parallel with the YOZ plane are collected from the tunnel point cloud, and the tunnel clearance inspection can be achieved by carrying out the clearance distance computation combined with the tunnel testing rack model.

### 2.2. Rail Linear Extraction and Establishment of Tunnel Clearance Coordinate System

The direct extraction of 3D rail based on point clouds requires the fine screening of tunnel point clouds. The rail point cloud is only reserved for fitting of the 3D rail point cloud, to obtain the 3D rail straight line and establish the tunnel clearance coordinate system.

#### 2.2.1. Rail Point Cloud Extraction

**Figure 4:**
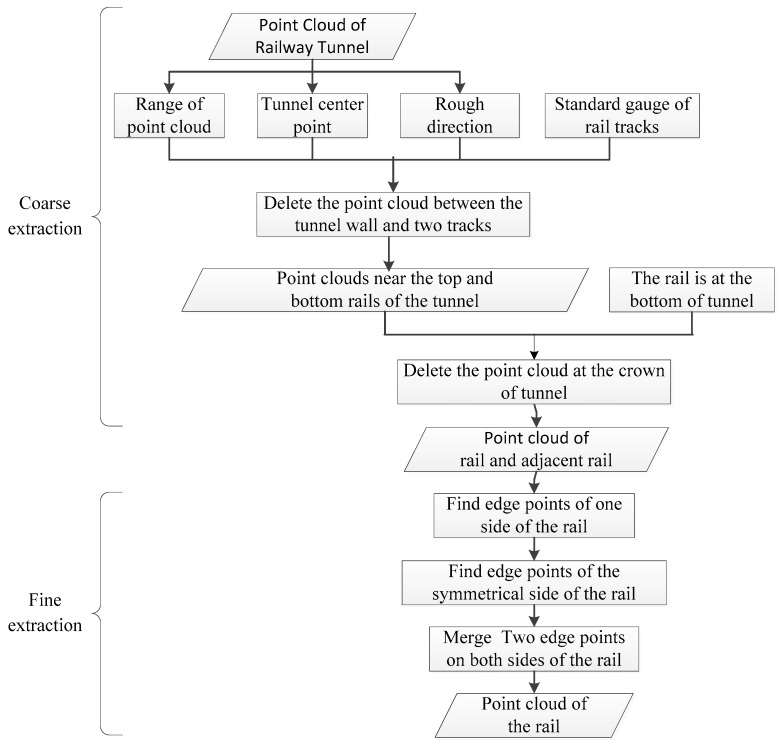
Flowchart of rail point cloud extraction.

The rail is laid in the middle of the tunnel, and there is a large space between the rail and the crown of the tunnel. The extraction of rail point cloud from a large amount of tunnel 3D laser point cloud includes two steps, coarse extraction and fine extraction, as shown in Figure 4.

(1) Coarse Extraction

The coarse extraction is to separate point cloud of two rails from the whole tunnel point cloud according the basic structure information of the tunnel. Firstly, the range of the point cloud can be calculated to obtain the center point coordinates of the tunnel and the rough alignment direction of rail. Secondly, the coarse rail lines can be reckoned with the center points and rough direction considering the gauge information regarding a redundancy about 0.2 m around the two rail lines. Then, the point cloud out of the two rail lines and between the two rail lines are deleted. Thus only the point cloud in the cube scope along the two rails is left. Thirdly, considering that the rails are located at the bottom of the tunnel, the differential elevation value of the points is used to eliminate the tunnel top point cloud. Through the coarse extraction, point cloud of two tracks and adjacent points are separated from the tunnel point cloud.

(2) Fine Extraction

**Figure 5:**
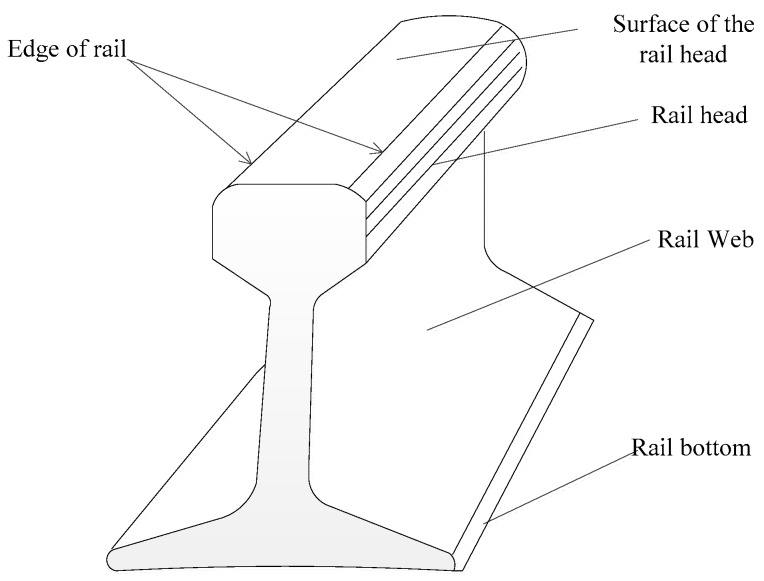
Rail section.

The rail cross section is I-shaped, composed of three parts, rail head, rail web, and rail bottom, as shown in Figure 5. Among the coarse extraction results of rail point cloud, the elevation values on the rail head point (or rail face point) on the left and right tracks are the maximum. The fine extraction of the rail track point cloud is the height from the coarse extraction results made by use of the rail surface, to achieve the fine extraction of the rail surface point through screening the maximum point.

Considering the entire rail alignment extension, the overall alignment of rail includes three cases, rising, falling and retaining. In case of falling or rising, the elevation of railway sleeper in the first half may be greater or less than the elevation of railway sleeper in the second half. Thus, the entire rail is then divided into several segments within 5 m to extract the surface point of the rail head one by one, which can avoid the effect of rail irregularities.

**Figure 6:**
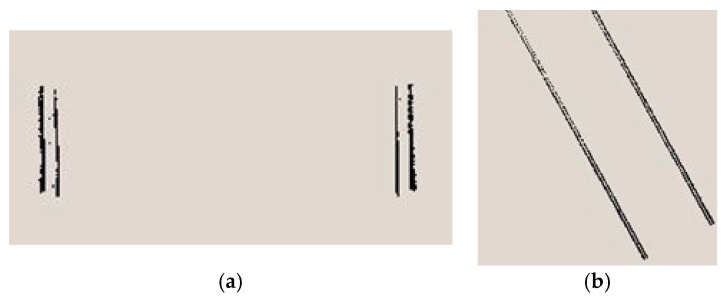
Fine extraction of rail point cloud. (**a**) Front View; (**b**) Top View.

As the rail surface is smooth enough to cause specular reflection, there are few laser points on the surface of rail head. Actually, the rail head only has laser points on both sides. There are edge points. Thus, the edge points of the both sides of the rail head are extracted to represent the rail. Then maximum elevation point in each segment is only the edge point on one side. To represent the complete rail, the edge points on both sides are needed. Therefore, after the local maximum elevation point on one side of the rail is obtained, the point corresponding to the other side can be obtained based plane distance and elevation difference constraint of the rail head. The points of the finally spliced segments are reflected by the edge points on both sides of the rail surface of the entire rail. Figure 6 shows the fine extraction result of rail point cloud.

#### 2.2.2. 3D Linear Fitting of Rail Line with Rail Point Cloud

**Figure 7:**
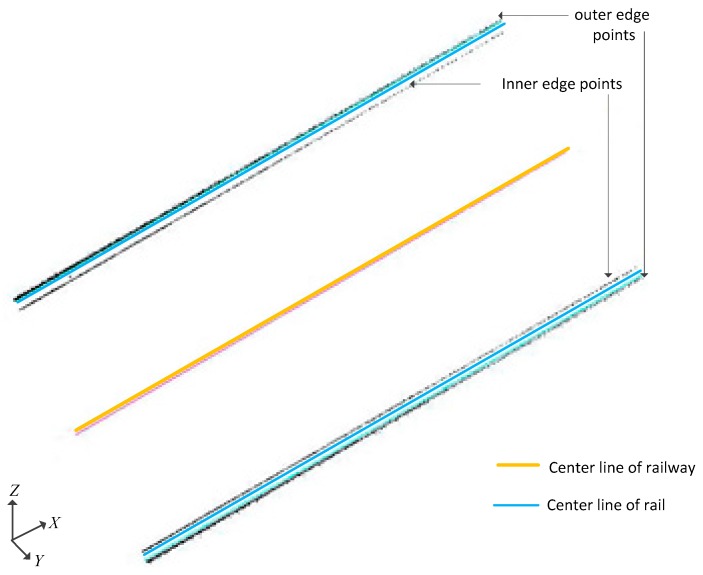
Rail line by 3D linear fitting using least square approach.

The space linear equation of the rail line is a continuous equation with six parameters, as Equation (1):

(1) $$\frac{x-x_{0}}{m}=\frac{y-y_{0}}{n}=\frac{z-z_{0}}{p}$$

where, $\left(x_{0},y_{0},z_{0}\right)$ is a point on the straight line, and (m,n,p) is the direction vector of the straight line. In this paper, after the coarse and fine extraction of the rail point cloud, two edge points of the rail head are obtained. The rail line can be calculated by the 3D linear fitting of the two edge points using the least square approach. Figure 7 is the result of 3D linear fitting of the rail lines with the edge points of the rail head.

In Figure 7, since the points on the outer edge of the rail head are more numerous than the points on the inner edge due to the scanning angle of MLS, the rail line by 3D linear fitting will be closer to the outside of the rail. Both rails must be parallel to each other in practice. Therefore, the two 3D rail lines 3D linear fitting should be parallel to each other. However, because of noise in laser scanning, point cloud extraction and 3D linear fitting have small errors. Thus, the two rail lines will not be completely parallel to each other, and there is a small angle between their direction vectors. To keep consistent with the actual rail structure and determine the clearance measuring coordinate system, the direction vector of two rail lines should be slightly modified. Thus, the average vector of the two rail lines are used as the direction vector of each rail line, to ensure that the two rail lines are parallel to each other. Finally, the centerline of two rail lines is taken as the centerline of the railway.

#### 2.2.3. Connection of the Segmented Rail Lines

The automatic extraction of rail in this paper is a process targeted at the rail in the segments which is approximately linear. The linear sections are sequentially connected after the extraction of the rail point cloud in the tunnel point cloud as well as the 3D linear fitting of rail lines, to obtain and reflect the continuous fold line. It is difficult for all rail segments to be seamlessly connected, so it is necessary to set some rules to connect rail segments into a continuous fold line as long as the precision is guaranteed. First, select a benchmark segment so that other segments move closer to it. To avoid the excessive cumulative error, it is suggested that the segment in the middle of the tunnel be used as a benchmark, rather than those on both ends. In this way, other segments on both ends of the benchmark get closer to it from afar, and the error arising from hereof will be halved compared with that from getting closer from one end.

The distance between the two fitting rail straight lines can be used as the restriction of checking the precision of the rail extraction and segmented rail connection. If the distance between two tracks in a given segment is largely deviating from the standard gauge, which is 1435 mm in China, it indicates that this section of rail extraction is in error. In case of rail segment cohesion, the segment with the distance between two track lines closest to the standard gauge shall be selected from multiple segments of tunnel center as the benchmark segment. The distance between two parallel lines is equivalent to the distance from one point in a straight line to the other. The direction vector of a straight line is (m,n,p), and a straight line passes through a point $\left(x_{0},y_{0},z_{0}\right)$, then the distance d from the point outside the straight line $P(x_{p},y_{p},z_{p})$ to the straight line can be calculated according to the Equation (2):

(2) $$d=\frac{\sqrt{|\begin{array}{c} y_{p}-y_{0}\\ n \end{array}{\begin{array}{c} z_{p}-z_{0}\\ p \end{array}|}^{2}\;+\;|\begin{array}{c} z_{p}-z_{0}\\ p \end{array}{\begin{array}{c} x_{p}-x_{0}\\ m \end{array}|}^{2}+|\begin{array}{c} x_{p}-x_{0}\\ m \end{array}{\begin{array}{c} y_{p}-y_{0}\\ n \end{array}|}^{2}}}{\sqrt{m^{2}+n^{2}+p^{2}}}$$

In this paper, as the linear fitted rail line is the center line of the track, it is not inside the rail edge, but between the inner edge and the outer edge. The distance between the two center lines includes standard gauge and the width of rail tread. Thus, the distance between the two tracks calculated by the Equation (2) is larger than the standard gauge. The distance empirical value obtained through a large number of testing data analysis is 1502 mm. The following is a simple analysis of the validity of this empirical value.

**Figure 8:**
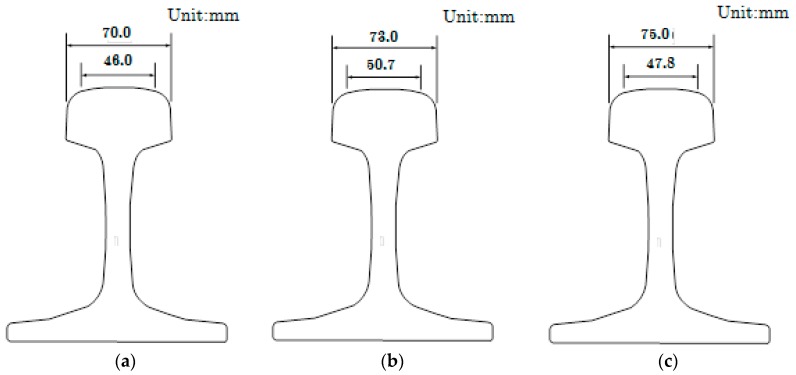
Dimensions of the rail tread. (**a**) 50 kg/m; (**b**) 60 kg/m; (**c**) 75 kg/m.

The type of rail is expressed as a mass kg per 1 m. The existing rail standards in China include three types, 75 kg/m, 60 kg/m and 50 kg/m. The greater the mass of the rail per meter, the greater the bearing load will be. To improve the carrying capacity of the line, the main lines are usually laid 75 kg/m or 60 kg/m heavy rails. The tread dimensions of the three rails are shown in Figure 8 [44,45]. The rail type data used in this paper is 60 kg/m, as shown in Figure 8, and the width of rail tread is 73.0 mm. Assuming that the rail straight line fitted by the rail point cloud is located right in the middle of the rail, the distance between the two tracks is 1508 mm (the sum of the standard gauge and tread width). Thus, the empirical distance of 1502 mm is more practical and reasonably guidance.

**Figure 9:**
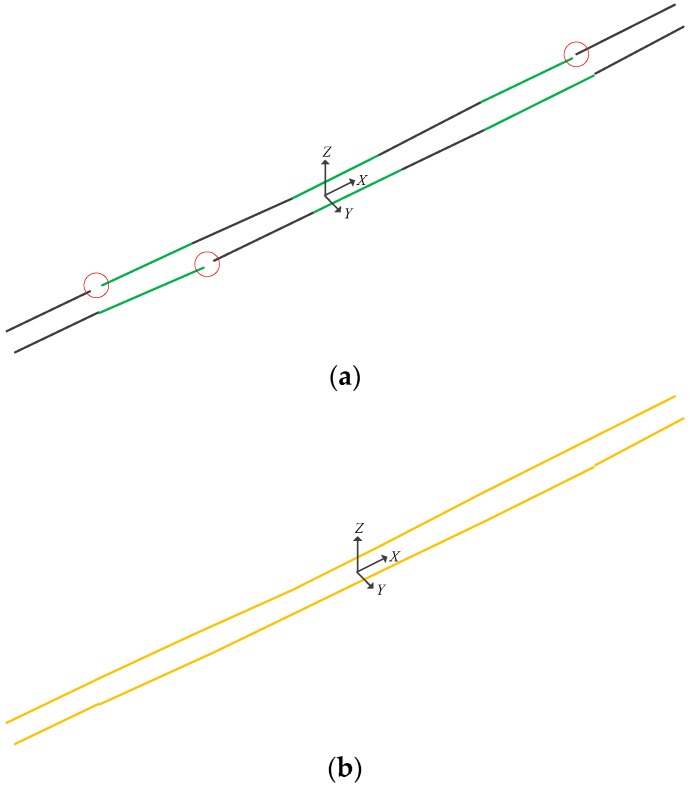
The connection result of all segmented rail lines. (**a**) Before connection; (**b**) After connection.

In order to connect all the segmented rail lines, a pair of segmented rail lines are selected as the connection benchmark from the center position of the tunnel center. The distance between the benchmark rail lines should be closest to 1502 mm. After the benchmark rail lines are selected, all the segmented rail lines can be connected from the benchmark lines to both the direction of the tunnel starting point and terminal point. First, the endpoint of the first adjacent segmented rail line should coincide with the nearest endpoint of the benchmark rail line. Then, keep the length and direction of first adjacent segmented rail line and move its other endpoint to make sure that the new rail line is parallel to the original rail line before this processing. The other sections are followed by analogy, and the seamless connection of the previous segments is achieved in sequence. The presented rail connection method will not change the rail direction of all segments, the distance between the rails in all segments should be consistent to ensure the continuity of the whole rail. The connection result of all segments are compared as shown in Figure 9.

In Figure 9, the two outer segments are segmented rail segments, and the middle line is the centerline of the rail. In Figure 9a, the adjacent segments are represented in different colors so that a minor gap exists between their joint, as indicated in red circle in the figure. After the connection of the segmented rail, all segments are seamlessly bonded, as shown in Figure 9b.

#### 3.2.4. Determination of Clearance Measuring Coordinate System

Tunnel clearance measuring coordinate system may be determined based on the following steps after two rail lines are obtained:

(1) Calculate rail centerline and determine axis *X*.

Two rail lines are parallel to each other and their direction vectors $\left(m_{1},n_{1},p_{1}\right)$ and $\left(m_{2},n_{2},p_{2}\right)$ meet the relationship in Equation (3):

(3) $$\frac{m_{1}}{m_{2}}=\frac{n_{1}}{n_{2}}=\frac{p_{1}}{p_{2}}$$

With known direction vectors $L_{1}(m_{1},n_{1},p_{1})$ and $L_{2}(m_{2},n_{2},p_{2})$ of two rail lines and points $M_{1}(x_{1},y_{1},z_{1})$ and $M_{2}(x_{2},y_{2},z_{2})$ respectively passing the two lines, direction vector L(m,n,p) obtained after the calculation of mean value of components of direction vectors $L_{1}$ and $L_{2}$ is the direction vector of center line of rail and point M(x,y,z) obtained after the calculation of mean value of coordinate components of points $M_{1}(x_{1},y_{1},z_{1})$ and $M_{2}(x_{2},y_{2},z_{2})$ is a point on the center line of rail. Equation (4) gives the relationship among three direction vectors and three points:

(4) $$\begin{array}{l} m=\frac{m_{1}+m_{2}}{2},n=\frac{n_{1}+n_{2}}{2},p=\frac{p_{1}+p_{2}}{2}\\ x=\frac{x_{1}+x_{2}}{2},y=\frac{y_{1}+y_{2}}{2},z=\frac{z_{1}+z_{2}}{2} \end{array}$$

After direction vector L(m,n,p) and point M(x,y,z) are obtained, the center line of railway may be determined according to Equation (1), i.e., the center line of track as well as axis X in the clearance measuring coordinate system.

(2) Determine axis *Y*.

The plane determined by two rail lines is the rail plane in tunnel clearance coordinate system, denoted as plane *A*. Axis *Y* in clearance coordinate system is perpendicular to axis *X* in plane *A*. The normal vector of plane *B* is the direction vector L(m,n,p) of the center rail line. The plane *B* is perpendicular to rail plane *A*. Two rail lines and plane *B* intersect at two points, the connecting line of which is axis *Y* and the center point of these two intersection points is the origin of coordinate system *O* for clearance calculation. The intersection points of rail line and plane *B* is calculated by linear parameter equation (as Equation (5)) and space plane equation (as Equation (6)):

(5) $$\left\{ \begin{array}{l} x=x_{0}+mt\\ y=y_{0}+nt\\ z=z_{0}+pt \end{array}\right.$$

where, $\left(x_{0},y_{0},z_{0}\right)$ is a point on the line, (m,n,p) is direction vector of the line and t is a parameter:

(6) $$A(x-x_{p})+B(y-y_{p})+C(z-z_{p})=0$$

where, $\left(x_{p},y_{p},z_{p}\right)$ is a point on the plane and (A,B,C) is normal vector of the plane.

The intersection points of rail line and plane *B* meets both linear parameter equation and space plane equation. Then, the parameter *t* of the intersection point can be obtained by combining Equations (5) and (6), as shown in Equation (7). Then, the coordinates (x,y,z) of the intersection point can be obtained by parameter *t* based Equation (5):

(7) $$t=\frac{A(x_{p}-x_{0})+B(y_{p}-y_{0})+C(z_{p}-z_{0})}{Am+Bn+Cp}$$

(3) Determine axis *Z*.

**Figure 10:**
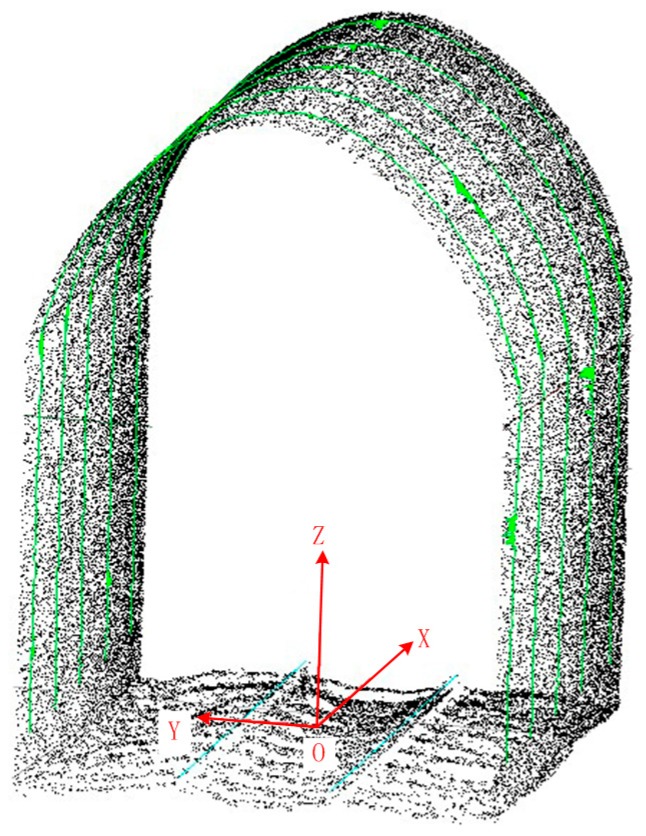
Tunnel clearance coordinate system.

The axis *Z* of the clearance measuring coordinate system meets the right-hand rule with axes X and Y in plane *B*, as shown in Figure 10. The green polyline in the figure is the cross section profile of tunnel at an interval of 1 m.

### 2.3. Profile Extraction Based on Clearance Coordinate System

The most commonly used method of tunnel clearance inspection is the cross section inspection method [46]. The tunnel cross section at certain interval is obtained in the clearance measuring coordinate system. Tunnel clearance inspection is realized through contrastive analysis on actual cross section profiles and design profiles. To determine whether the tunnel space can guarantee the driving safety of trains, it is judged by calculating the distance between locomotive model and rail tunnel cross section profile.

In the clearance measuring coordinate system shown in Figure 10, if a cross section perpendicular to the plane *XOY* is selected from the tunnel point cloud directly, the number of points on a cross section might be too low to show the complete cross section profile due to the limitation of point cloud density. A triangulated irregular network (TIN) is widely used in representation of the physical surface. A TIN comprises a triangular network of vertices, known as mass points, with associated coordinates in three dimensions connected by edges to form a triangular tessellation[47,48]. Thus, a TIN is introduced to overcome the clearance inspection problem with the discrete point cloud, which can be built with the tunnel point cloud, or presenting the tunnel cross section via the point of intersection of TIN and cross section. The specific steps are as below:

Step 1: establishment of TIN for the tunnel point cloud. This paper introduces TIN into the generation of continuous tunnel model and describes three-dimensional tunnel with TIN. Due to large data volume of tunnel point cloud, to reduce the amount of calculation in establishment of TIN, tunnel point cloud are first selected. The clearance inspection only needs to calculate along the cross section. In order to improve the robustness, we use points on a narrow stripe of the cross section. These can also reduce the amount of calculation for tunnel TIN building. Thus, only points on the tunnel wall and tunnel top within a certain scope along the cross section are reserved to generate the *TIN* of the cross section.

Step 2: obtainment of the tunnel cross section based on TIN. After the clearance measuring coordinate system is determined, plane *B* of the cross section which is perpendicular to the rail line can be obtained. Assuming that $\left(x_{p},y_{p},z_{p}\right)$ is the datum point of cross section on axis *X* in clearance measuring coordinate system, the plane *B* of cross section can be determined by the direction vector (A,B,C) of axis X as Equation (8):

(8) $$A(x-x_{p})+B(y-y_{p})+C(z-z_{p})=0$$

The point of intersection of each side of each triangle and cross section in *TIN* can be calculated by calculating the point of intersection with the plane by parametric equation of line. Such intersection points are sorted by their coordinate *z*. The profile of tunnel cross section is obtained after ordered points are connected into lines.

### 2.4. Clearance Calculation

**Figure 11:**
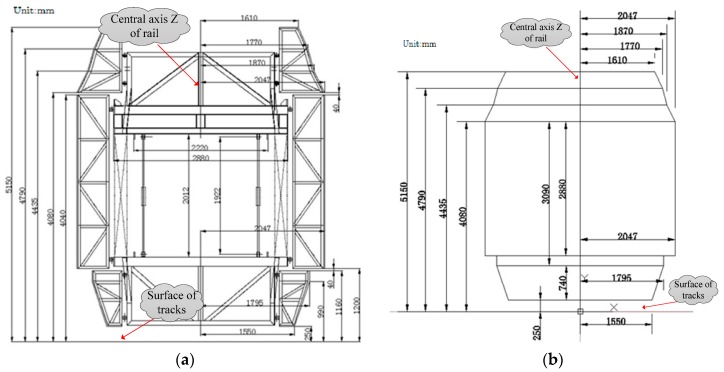
Standard Clearance test frame Model of Rail Tunnel. (**a**) Standard clearance frame model; (**b**) Simplified model of *SCFM*.

Figure 11a shows standard clearance test frame model (SCFM) of a rail tunnel and its dimensions. Figure 11b shows simplified clearance test frame model and its dimensions.

**Figure 12:**
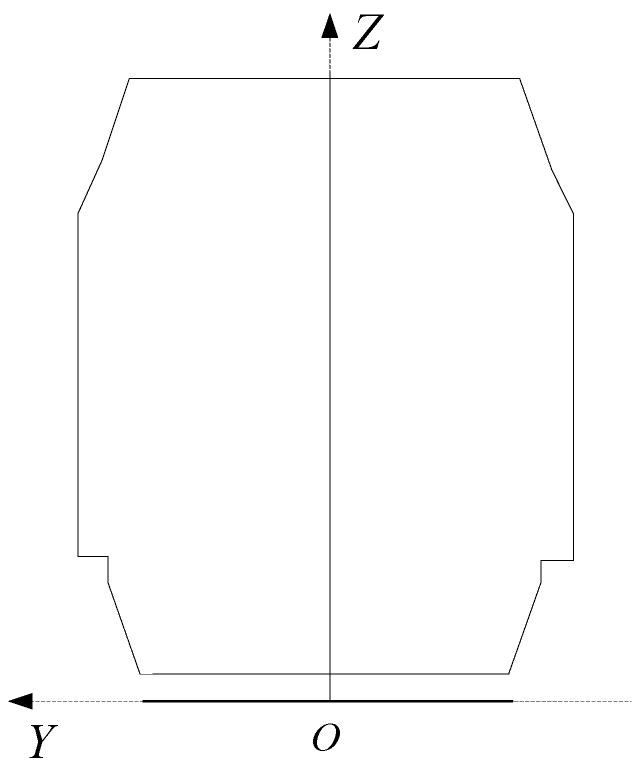
Unification of clearance test frame model and clearance coordinate system.

Clearance calculation must be conducted through unification of standard clearance test frame model of tunnel into clearance measuring coordinate system. Specific steps are as below:

Step 1: Place the standard clearance test frame model in ZOY horizontal plane and make the mean axis of the model overlap with axis *Z* and bottom axis of the model overlap with axis *Y*, as shown in Figure 12.

Step 2: clearance inspection calculation. The railway tunnel clearance inspection is realized by calculating the distance between polyline of tunnel cross section profile and standard clearance test frame model. This paper divides clearance test frame into top, side and bevel types based on its structure. The significance of its clearance inspection is shown as below:

(1) Clearance calculation between the cross section profile and the top of tunnel, the side of tunnel

Divide the cross section into tunnel top and tunnel wall with certain height according to the tunnel design material, calculate the distance between cross section and the corresponding part of clearance test frame respectively (Figure 12). The distance represents the clearance between cross section profile and the top of tunnel, the side of tunnel respectively. This distance can be used to guide tunnel widening or rail track alining.

(2) Clearance calculation between overhead contact line (OCL) and the top of clearance test frame

**Figure 13:**
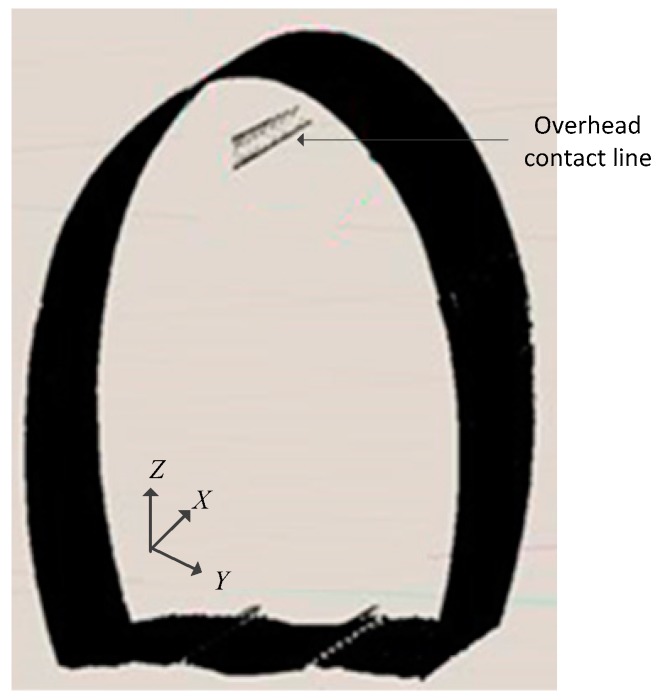
Point cloud of tunnel and OCL.

OCL is generally near the top of centerline of track (Figure 13). It is only possible to scrape the top of clearance test frame. Therefore, it is only necessary to calculate its distance to the top of clearance test frame. Such distance may be used for conducting sag analysis on OCL and guiding the maintenance of OCL to guarantee driving safety of train.

To improve the reliability of the clearance calculation, data within a certain scope of the cross section profile along the direction of axis *X* are generally selected to constitute the cross section zone for calculating the interval with the clearance test frame. Considering the density of the point cloud, this paper defines this scope as 0.01 m, respectively, before and after the cross section profile along the direction of axis *X*.

## 3. Experimental Result and Discussion

### 3.1. Experiment Place and Data

**Figure 14:**
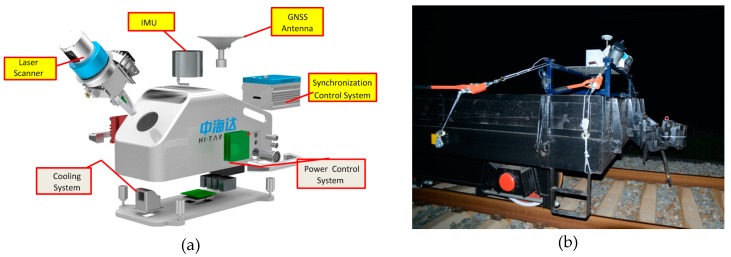
Mobile Laser Scanner (MLS) for railway tunnel point cloud collection. (**a**) Components of MLS; (**b**) MLS on platform trailer for field data collection.

Experiment data in this paper are point clouds of the Shuanghekou Tunnel of the Chengdu-Kunming Line from a mobile three-dimensional laser scanning system (as shown in Figure 14). This system is composed of a three-dimensional laser scanning sensor, GPS, inertial measurement unit (IMU), mileage coder and control storage cell and placed on a track trolley for data acquisition with pushing.

**Figure 15:**
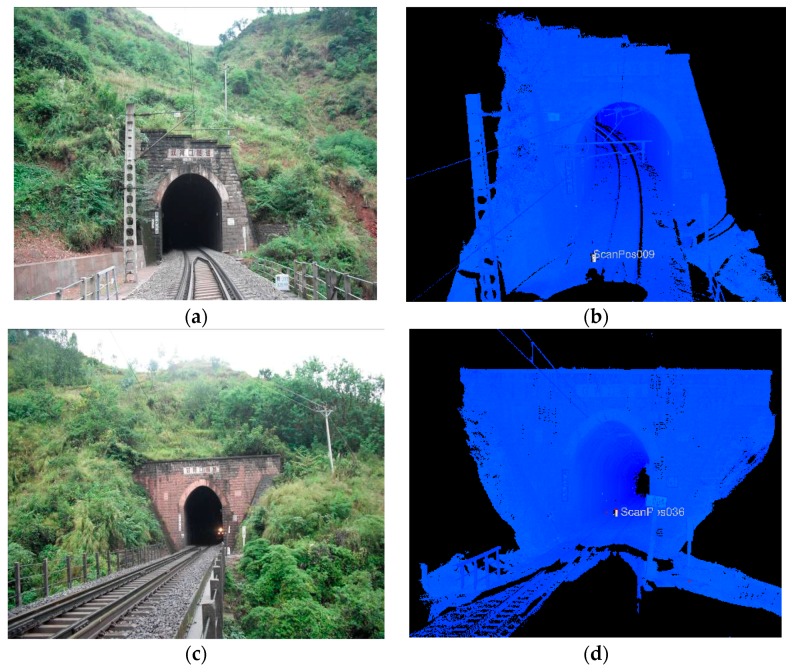
Picture and point cloud of tunnel entrance and exit. (**a**) Picture of tunnel entrance; (**b**) Point cloud of tunnel entrance; (**c**) Picture of tunnel exit; (**d**) Point cloud of tunnel exit.

The density of the tunnel point cloud obtained is better than 1 point/cm^2^ and scanning precision at 10 m is lower than 1 mm. The Shuanghekou Tunnel is located between Ganluo and Nanergang on the Chengdu-Kunming Line with an overall length of 1889.5 m. Figure 15 shows the image and point cloud of the tunnel entrance and exit.

This paper selects data of three sections of the tunnel for experiment and analysis on tunnel clearance inspection, i.e., tunnel entrance, tunnel middle and tunnel exit. Table 1 describes the data set.

For the three data sets in Table 1, a clearance coordinate system is established and clearance inspection analysis is conducted with the method mentioned in this paper. The precision of establishment of clearance coordinate system may be expressed by the fitting precision of rail line. For clearance inspection, the value of clearance of different types is respectively calculated according to Section 2.4.

### 3.2. Experimental Result Analysis

#### 3.2.1. Precision Result of Clearance Coordinate System

Fitting precision of rail line in each section in data sets of three different positions is respectively subject to statistics. This paper uses the mean value of distance between each rail point and fitting rail line as fitting precision index of rail line, i.e., precision of clearance coordinate system, as shown in Table 2.

**Figure 16:**
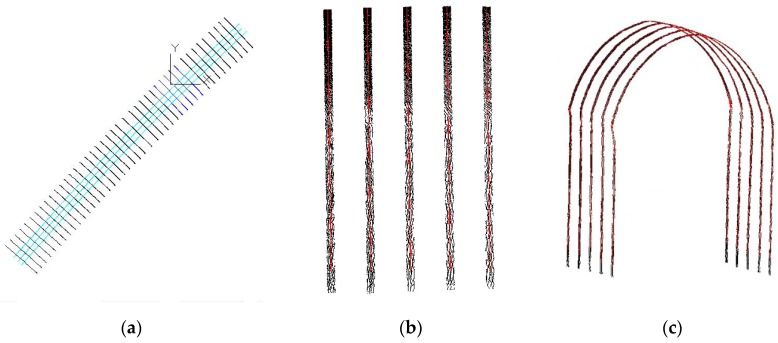
Tunnel cross section profile. (**a**) Top view; (**b**) Side view; (**c**) Phantom view.

According to Table 2, points of rail lines are extracted from the tunnel point cloud data. The fitting precision of three-dimensional for rail line with point cloud in different positions and sections is better than 0.03 m, i.e., the precision of tunnel clearance coordinate system is better than 0.03 m, which meets the requirement of clearance inspection. The cross section profile is extracted from tunnel TIN at an interval of 1 m based on clearance measuring coordinate system determined via rail line, as shown in Figure 16:

#### 3.2.2. Calculation of Clearance between Cross Section Profile and the Top of Clearance Test Frame and Side

**Figure 17:**
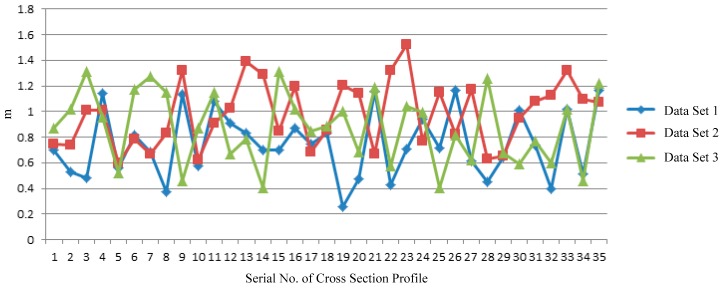
Clearance inspection result of tunnel wall.

Figure 17 shows the result of clearance calculation between cross section profile in three different positions and the top of clearance test frame and side.

According to Figure 17, minimum clearance distance between tunnel cross section profile and the top, the side of clearance test frame, is respectively 0.258 m, 0.598 m and 0.401 m, indicating that tunnel wall and top do not cross the border and are within safe distances.

#### 3.2.3. Clearance Calculation of OCL and the Top of Clearance Test Frame

**Figure 18:**
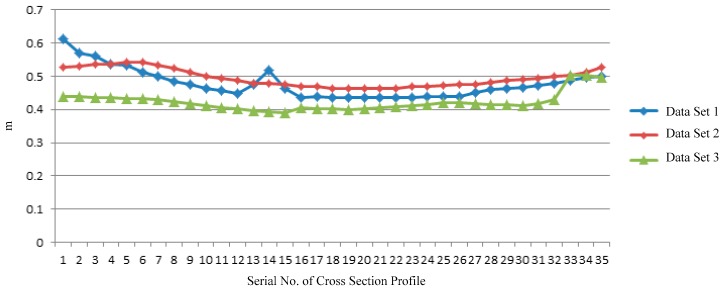
Clearance distance between OCL and the top of clearance test frame.

Figure 18 presents the result of clearance calculation between OCL and the top of clearance test frame in three different positions.

Figure 18 reflects sag degree of OCL indirectly. The smaller the distance is, the greater the sag will be. Actual OCL sag is consistent with this clearance line chart. Minimum distance between OCL and the top of clearance test frame is respectively 0.435 m, 0.462 m and 0.390 m. This clearance can guarantee safe passage of a train. As Figure 17 and Figure 18 have shown, the minimum distance are more than 10 times the precision of the clearance coordinate system in Table 2. This means the errors of 3D linear fitting can be ignored and the clearance inspection result is reliable.

### 3.3. Discussion

Though this paper realizes railway tunnel clearance inspection based on high-precision and high-density point cloud obtained with mobile three-dimensional laser scanning, three-dimensional laser point clouds have a large data volume and such process as segmentation of tunnel point cloud, extraction of point cloud in each section of rail and establishment of TIN for cross section point cloud involve a lot of point cloud reading and writing throughput. It is necessary to further improve the efficiency of point cloud processing. For example, it is a feasible solution to index point clouds with hierarchy of segmentation and conduct parallel and accelerated processing based on GPU. In addition, the method in this paper does not apply to online clearance inspection. To establish online clearance coordinates in combination with real-time GPS/IMU integrated navigation, it is necessary to consider the configuration of the sensor in three-dimensional laser mobile scanning system. For example, it is a technical approach to resolve real-time clearance inspection by installing three-dimensional laser scanner parallels to clearance coordinate system, correcting it with real-time GPS/IMU integrated navigation and attitude and unifying it into the same clearance coordinate system [49].

Current clearance inspection is a kind of safety check before railway operation. The clearance inspection can be implemented after on-site data collection through post-processing and data analysis. The proposed clearance inspection approach is not sensitive to the time for the line extraction is based on the segmented point cloud. The clearance inspection calculation is implemented with the cross section profile with low computational complexity. However, the real time clearance inspection will increase the railway operation efficiency, and the next clearance inspection device and approach should take more consideration of the data processing.

Moreover, this paper only defines one clearance test frame model. If multiple types of clearance test frame models are added and the distance between tunnel cross section profile and a certain clearance test frame model is calculated selectively, tunnel clearance inspection for different types of locomotives can be realized.

## 4. Conclusions

Tunnel clearance inspection is vital for guaranteeing the safe operation of locomotives and improving the traffic capacity and cargo capacity. This paper introduces a three-dimensional laser mobile scanning system into the railway tunnel clearance calculation and inspection field, and puts forward a processing flow and technical frame for railway tunnel clearance inspection based on three-dimensional laser point clouds. According to the long and narrow structure of rail tunnel, three-dimensional laser point clouds of tunnels are subjected to segmented pretreatment along the direction of the railway line, to guarantee a straight line of rail in each section. Linear segments of rail are extracted from laser point clouds on this basis and a clearance measuring coordinate system is determined with the center line of two rails and the plane of the rails. TIN is established with the tunnel point cloud. Meantime, the point of intersection of TIN and the cross section will be calculated. Tunnel cross sections with equal intervals are thus produced. Finally, tunnel clearance inspection is realized by calculating the distance between the cross section profile and the tunnel clearance test frame with the tunnel cross section profile, combined with a tunnel testing-rack clearance calculation method. By taking the Shuanghekou Tunnel of the Chengdu-Kunming Railway as an example, when the clearance inspection is carried out by the method mentioned herein, its precision can reach 0.02 m, and difference types of clearances can be effectively calculated. This method thus has wide application prospects.

## Figures and Tables

**Table 1:** Experiment dataset.

Data set	Position	Radius of Curvature (m)	Length (m)	Section	Number of Points
1	Tunnel entrance	500	35	7	23,253,815
2	Tunnel middle	Greater than 1000	35	7	27,367,654
3	Tunnel exit	Greater than 1000	35	7	30,127,334

**Table 2:** Precision of clearance coordinate system (Unit: m).

	Fitting Precision	Data Set 1		Data Set 2		Data Set 3	
Section No.		Left Rail	Right Rail	Left Rail	Right Rail	Left Rail	Right Rail
1		0.025	0.030	0.020	0.024	0.021	0.011
2		0.012	0.011	0.020	0.013	0.029	0.029
3		0.008	0.013	0.016	0.011	0.018	0.017
4		0.010	0.008	0.013	0.016	0.016	0.015
5		0.019	0.021	0.012	0.008	0.015	0.020
6		0.009	0.008	0.009	0.008	0.018	0.007
7		0.008	0.008	0.012	0.021	0.019	0.018
Mean value		0.013	0.014	0.014	0.014	0.019	0.017
